# Author Correction: Segment Anything for Microscopy

**DOI:** 10.1038/s41592-025-02745-9

**Published:** 2025-06-10

**Authors:** Anwai Archit, Luca Freckmann, Sushmita Nair, Nabeel Khalid, Paul Hilt, Vikas Rajashekar, Marei Freitag, Carolin Teuber, Melanie Spitzner, Constanza Tapia Contreras, Genevieve Buckley, Sebastian von Haaren, Sagnik Gupta, Marian Grade, Matthias Wirth, Günter Schneider, Andreas Dengel, Sheraz Ahmed, Constantin Pape

**Affiliations:** 1 Georg-August-University Göttingen, Institute of Computer Science, Goettingen, Germany; 2 German Research Center for Artificial Intelligence, Kaiserslautern, Germany; 3 RPTU Kaiserslautern-Landau, Kaiserslautern, Germany; 4 Department of General, Visceral and Pediatric Surgery, University Medical Center Göttingen, Göttingen, Germany; 5 Ramaciotti Centre for Cryo-Electron Microscopy, Monash University, Melbourne, Victoria Australia; 6 Georg-August-University Göttingen, Campus Institute Data Science, Goettingen, Germany; 7 CCC-N (Comprehensive Cancer Center Lower Saxony), Göttingen, Germany; 8 Department of Hematology, Oncology and Cancer Immunology (Campus Benjamin Franklin), Charité Universitätsmedizin, Berlin, Germany; 9 German Cancer Consortium (DKTK), Heidelberg, Germany; 10 Max Delbrück Center, Berlin, Germany; 11 Clinical Research Unit 5002, KFO5002, University Medical Center, Göttingen, Germany; 12 Cluster of Excellence ‘Multiscale Bioimaging: from Molecular Machines to Networks of Excitable Cells’ (MBExC), Georg-August-University Göttingen, Göttingen, Germany

**Keywords:** Image processing, Software

Correction to: *Nature Methods* 10.1038/s41592-024-02580-4, published online 12 February 2025.

In the version of the article initially published, Melanie Spitzner, Constanza Tapia Contreras, Marian Grade, Matthias Wirth and Günter Schneider were missing from the author list and have now been added. Additionally, Constantin Pape’s second affiliation (Cluster of Excellence ‘Multiscale Bioimaging: from Molecular Machines to Networks of Excitable Cells’ (MBExC), Georg-August-University Göttingen, Göttingen, Germany) was missing and has now been added. These corrections have been made to the HTML and PDF versions of the article.

