# Supplementary material for: Segment Anything for Microscopy
Source: Nat Methods. 2025 Feb 12;22(3):579–91. doi: 10.1038/s41592-024-02580-4 (PMC11903314; doi:10.1038/s41592-024-02580-4)
Supplement: Supplementary file 1 — Supplementary text on software implementation and user study, links to Supplementary Videos 1–5, Supplementary Tables 1 and 2, Supplementary Figs. 1–4 and Supplementary Information-only references. [file 41592_2024_2580_MOESM1_ESM.pdf]

nature methods

Supplementary information

https://doi.org/10.1038/s41592-024-02580-4

Segment Anything for Microscopy

In the format provided by the authors and unedited

Supplementary Information

This document contains supplementary material for our manuscript “Segment Anything for Microscopy”. It contains more extended descriptions of our software implementation and our user studies that did not fit into the methods section. In addition, it lists the supplementary videos that contain tutorials for or napari plugin. We also provide supplementary tables that list all the data used in our study and supplementary figures that give an overview of additional experiments we have conducted.

Software Implementation

We extend the core functionality of SAM in multiple ways to enable efficient interactive and automatic segmentation for multidimensional data:

- We implement precomputation of the image embeddings. This step takes the bulk of computation time and is independent of the user-provided annotations, see Fig. 5 a for time measurements. We also support caching the embeddings to file, so that they do not have to be recomputed upon restarting the annotation tool for a given input. This is of particular importance for volumetric or time series data. Image embeddings can also be precomputed on separate resources with a GPU (*e.g.* local workstation, on-premise computer cluster or cloud) and then be copied to the laptop used for annotation.
- We implement tiled computation for embeddings as well as interactive and automatic segmentation. When using this feature the embeddings are computed for overlapping patches in the input image. In interactive segmentation the tile that best matches the current annotations is chosen and the mask is predicted for it; the overlap ensures that objects that are part of multiple tiles can be segmented and the overlap size has to be chosen accordingly. Tiled embeddings are also precomputed and can be saved to file.
- For automatic segmentation (both AMG and AIS) we separate the computationally expensive steps (computing masks for grid points / applying the decoder) from the post-processing steps (filtering masks / running seeded watershed). Due to this design users can interactively adjust the instance segmentation parameters and find the best settings for their images. It also allows precomputation of the state for automatic segmentation, which can be stored together with the embeddings. This feature is also used to efficiently perform the grid search mentioned in the previous section.
- We extend SAM’s interactive segmentation functionality to multiple dimensions (volumetric or 2D + time) by projecting input annotations derived from a given object to adjacent slices / frames. We derive (low-resolution) input masks, the bounding box and point annotations and can present SAM with any combination of these input annotations. The best combination depends on the model type and application; we investigate this in Ext. Data Fig. 9 a and set the best parameters according to our experiments for a given model in the plugin for interactive segmentation automatically. For tracking we project the annotations based on a linear motion model to better follow objects with a directed movement (see below for details). We use the IOU between the predicted masks in

adjacent slices / frames as a stopping criterion to prevent following objects that are wrongly segmented or not present anymore (e.g. because the structure being segmented has stopped or the cell being tracked leaves the frame). This functionality also supports interactive correction: after adding annotations in a slice / frame the whole object / track will be recomputed accordingly. For tracking we support cell divisions by individually tracking the cells after a division event.

- We implement automatic 3D segmentation by segmenting the image volume slice by slice (using either AMG or AIS) and then merging objects across slices based on a multicut graph optimization problem[66] derived from object overlaps. To reduce segmentation artifacts due to the lack of 3D context we apply a closing operation before this step to fully segment objects for which the slice-by-slice segmentation is missing for some intermediate slices. We evaluate interactive and automatic multidimensional segmentation in Ext. Data Fig. 9.

Based on this functionality, we implement a tool as a napari plugin. It is made up of five different widgets: the 2D (i) and 3D (ii) annotation widgets, the tracking annotation widget (iii), the image series annotation widget (iv) and the finetuning widget (v). We use existing napari functionality to implement them whenever possible. The napari *label layer* is used to represent object masks from interactive and automatic segmentation. This enables using the features of this layer, such as importing segmentations from file, manually editing objects or saving the layer to file. The *point layer* is used to represent point annotations and the *shape layer* for box annotations. For the latter case we support *rectangle* annotations, which are directly mapped to boxes, as well as *ellipse* and *polygon* annotations, which are used to derive the corresponding bounding box and low resolution mask as input for SAM. The additional GUI elements of our tool are generated with magicgui[53], which generates menus from type-annotated function signatures and with PyQT for more complex user interfaces. The interactive segmentation functionality is implemented with four layers: the *point_prompt* and *prompt* layers are used for the user-provided annotations. The *current_object* layer contains the objects that are currently being annotated, and the *commited_objects* layer contains objects that have already been annotated. Once the user is done with the current object(s) they press a button to move it (them) to *commited_objects*. The tool precomputes the image embeddings for the complete image data, so that only prompt encoder and mask decoder have to be reapplied when the annotations change, enabling response times of below one second on a consumer laptop. They also all support tiled embeddings to enable annotation of large image data.

The 2D and 3D annotation widgets (i, ii) support automatic instance segmentation, which is implemented via either AMG or AIS, depending on the selected model. Its outputs are stored in the *auto_segmentation* layer and its state is precomputed to enable interactively changing parameters as described above. When using box annotations the 2D annotation widget (i) can segment multiple objects at a time, one per box, and we also implement a *batched* annotation mode that enables segmenting multiple objects with point annotations, one object per positive point. Interactive correction of an object with point or point and box annotations is only possible for a single object at a time. In the 3D annotation widget interactive annotation is supported only for one object at a time. The tracking annotation widget (iii) supports interactive annotation with

dividing objects by extending the point annotations with properties to mark division events and to differentiate separate cells (mother and daughter cells) within a lineage. Otherwise it supports similar features to the 2D and 3D widget, without support for automatic segmentation and restricted to tracking a single lineage at a time. To follow moving objects we use a simple motion model for projecting prompts to the next frame in interactive tracking by shifting the prompts by the object's current motion vector. We compute this motion vector as the weighted moving average of the object's center of mass displacement between frames. The weight used for the moving average is controlled by the *motion_smoothing* parameter, which can be set in the user interface. We also implement an annotation widget for a series of 2D or volumetric images (iv), e.g. for multiple images stored in a folder. This widget precomputes the embeddings for all images, and then enables the users to iteratively annotate them, automatically saving the segmentation results per image to a folder. It enables high-throughput annotation of images. The finetuning widget (v) enables training SAM on user data and offers the settings for the different hardware configurations we have investigated in Ext. Data Fig. 10 and Supp. Fig. 3. The individual settings can also be modified. Finetuning is also supported via scripts so that users with computational experience can run it on resources with more powerful GPUs, like a compute cluster or in the cloud.

Our contributions are implemented in the µSAM python library. To enable programmatic use we implement this library in a modular fashion, in particular to enable using most of the functionality without requiring importing napari and starting a GUI. We use scipy[54] and scikit-image[55] as well as our own library *elf* (https://github.com/constantinpape/elf) to implement various image processing logic needed for interactive and automatic segmentation. We provide an extensive documentation for both the python library and GUI online.

User Study

The first user study was performed by five different annotators, the two others by a single annotator each. All annotators are experienced with the respective type of data and segmentation / tracking problem. They familiarized themselves with µSAM and the reference tools before starting the experiments to measure annotation times and quality. They all used consumer grade laptops for their experiments. For measuring the annotation times we exclude long running operations that don't require manual intervention, such as computing the image embeddings for µSAM, training models for µSAM and CellPose and computing supervoxels and the region graph for ilastik carving. These times are reported separately for the first user study below. Note that our goal is to give a comparison of data annotation with µSAM and widely used tools for a given annotation task. These experiments have some degrees of freedom, but our goal was to provide as fair comparisons as possible.

For the user study of the 2D annotation tool we use an internal dataset of organoids imaged with a Sartorius Incucyte microscope in brightfield microscopy. The organoids were treated with different drugs that inhibit their growth, including images from control experiments that were not treated. A typical image contains around 100 organoids, though this number varies depending

on the treatment and the time of imaging. We use a total of 12 images for the annotation experiments and measure three different quantities: the average annotation time per organoid (“Annotation Time”), the quality of annotations when compared to consensus annotations via mean segmentation accuracy (“mSA (Ann)”, see below for details on consensus annotations) and the generalization quality (“mSA (Test)”), measured by mean segmentation accuracy of the respective model evaluated on a separate test split with 15 images and associated ground-truth segmentations. To obtain these measurements for the different methods reported in Fig. 6 a we proceed as follows:

- For “Manual” we annotate 3 images manually using the paint functionality of the napari label layer. We don’t report “mSA (Test)” here because this method does not involve a segmentation model.
- For “µSAM (Generalist)” we annotate 3 images (same as “Manual”) using the LM Generalist model. We first segment the organoids automatically using AIS, remove wrongly segmented objects and then segment the remaining objects interactively. Note that this model produces too large segmentation masks in interactive segmentation, likely due to a bias introduced by the training data. We did not further correct these annotations, leading to fast annotation times but relatively low annotation quality, see also Fig. 6 a. For “mSA (Test)” we report the performance of the LM generalist model on the test set.
- For “µSAM (Default)” we annotate 6 images (superset of “Manual”). We proceed as for “µSAM (Generalist)”, but use AMG for automatic segmentation instead of AIS. The values for “mSA (Test)” are computed by finetuning a model on the 6 annotated images and applying the resulting model to the test set. We start the finetuning from the LM Generalist model, as it has a pretrained segmentation decoder. We find that its bias to enlarge object masks of this model is gone after finetuning, see also next point. Note that the segmentation quality of the default model with AMG itself is not reported in the table; it achieves an mSA of 0.39 on the test set.
- For “µSAM (Finetuned)” we use the finetuned model obtained from “µSAM (Default)” and annotate 6 different images following the usual procedure (automatic segmentation followed by correction via interactive segmentation). The values for “mSA (Test)” are derived from a model that was trained on the 12 images annotated with “µSAM (Finetuned)” and “µSAM (Default)”. We chose this approach to obtain the model that would result from a typical annotation workflow, where the user first annotates some images with a pre-trained model, then finetunes a model, annotates further images with it and finetunes another model on all annotations.
- For “CellPose (Default)” we annotate 3 images (same as “Manual”) with the *cyto2* model in the CellPose GUI. We first automatically segment the organoids and correct the segmentation using the painting functionality of the CellPose GUI.
- For “CellPose (HIL)” we use the human-in-the-loop training functionality of the CellPose GUI to finetune an image on the so far annotated images after each newly annotated image. We do this for 6 images (same as “µSAM (Default)”) and for each image first run automatic segmentation followed by manual correction. We start from the *cyto2* model. For a new image we use the latest model. However, for some cases the model obtained via finetuing overfits, leading to a bad segmentation results. In these cases we use the

cyto2 model to generate the segmentation instead. The “mSA (Test)” is obtained by applying the last model from in-the-loop training to the test set.

- For “CellPose (Finetuned)” we use the model obtained after “CellPose (HIL)” to annotate the 6 new images (same as “µSAM (Finetuned)”) without further training with the usual approach of automatic segmentation and correction. The “mSA (Test)” is obtained from a model trained offline on the combined 12 annotated images from “CellPose (HIL) and CellPose (Finetuned)”, following the same logic as described for “µSAM (Finetuned)”.

For all µSAM models we use the image series annotation widget and models with ViT-B image encoder. The consensus annotations were obtained by averaging the foreground pixels of all annotations for a given image, treating all pixels that were annotated with a fraction of over 0.6 as true foreground. After this, we compute affinities for all annotations, average these and run mutex watershed[49] constraint to the foreground mask to obtain an instance segmentation. This result is then manually proof-read by one of the annotators. In addition to the measurements in Fig. 6 a, we have also tracked the average runtimes of preprocessing and training steps:

- Preprocessing with “µSAM (Default)” takes 88.2 +- 55.1 seconds per image.
- Preprocessing with “µSAM (Generalist)” and “µSAM (Finetuned)” takes 12.7 +- 8.5 seconds per image.
- Training the model used in “µSAM (Finetuned)” takes 6.8 +- 2.0 hours in total. Note that training is performed once after all images are annotated.
- Training for “CellPose (HIL)” takes 535.8 +- 6.8 seconds per image. Note that training is performed after each newly annotated image.

The evaluation code for this user-study can be found at https://github.com/computational-cell-analytics/user-study-v3.git.

For the user study of the 3D annotation tool we use an internal dataset of tissue from the fruitfly larva brain imaged in volume EM. We make use of two small blocks. Each contains ca. 40 nuclei and one of them contains annotations that were also created with µSAM. This block is used for model finetuning, the other one is used to measure the annotation times as follows:

- For “µSAM (Default)” we annotate it with the default SAM ViT-B model. We use interactive annotation for all nuclei in the volume. Note that we tried using the finetuned EM model for mitochondria and nuclei here, but it yielded slightly worse results, most likely due to the mismatch of resolution and difference in appearance, including imaging artifacts, of this data compared to the generalist’s training data.
- For “µSAM (Finetuned)” we finetune the default SAM ViT-B model on the other block (with ground-truth) and then use it for annotation. In this case we first automatically segment the volume using AIS, remove wrong segmentations and then interactively segment the remaining nuclei. The training block is of the same size as the annotation block; generating this training data took around one hour with µSAM.
- For “ilastik” we use the ilastik carving workflow to interactively annotate the training data. Carving uses a seeded graph watershed based on supervoxels that are derived from an edge filter applied to the image volume. We first find good choices for edge filter, supervoxel generation and bias (important parameter) and then annotate the nuclei interactively with carving.

We report the average annotation time per object in the annotated block. We visually checked that all nuclei were segmented in the three annotation results. It was not possible to segment the nuclei correctly on the pixel level with ilastik carving due to errors in the underlying supervoxels, resulting in rough segmentation boundaries. The segmentation quality from µSAM is better (see Fig. 6 c), but some pixel-level errors remain in slices with imaging artifacts. These could be corrected by manual painting, which was not done for the user study and would increase annotation times.

For the user study of the tracking annotation tool we make use of two dataset from Schwartz et al.[41], which contain timeseries of nuclei imaged in fluorescence microscopy and ground-truth annotations for segmentation and tracking. To make the tracking problem more challenging we use every 3rd frame; we found that using every frame leads to a simple problem that could be solved almost perfectly by tracking via overlap. We use one of the datasets for the annotation experiments and the other for finetuning. To measure the annotation times for Fig. 6 c we proceed as follows:

- For “µSAM (Default)” we annotate each lineage, corresponding to the tracks of a cell from the initial frame and its daughter cells, with the default ViT-L model using the interactive annotation functionality of µSAM.
- For “µSAM (LM Generalist)” we proceed similarly to “µSAM (Default)” but use the LM generalist ViT-L model.
- For “µSAM (Finetuned)” we finetune the ViT-L LM Generalist on the separate dataset and then annotate the same dataset as in the other experiments. The dataset used for finetuning is of the same size as the annotation dataset.
- For “TrackMate (Stardist)” we annotate the dataset with TrackMate, starting from an initial nucleus segmentation provided by StarDist. We use the StarDist integration of TrackMate to obtain it. TrackMate then computes an automated tracking result, which is then corrected with TrackMate’s in-built correction functionality.

We report the average time per track, corresponding to the track of a single cell ending at a division event or at the end of the timeseries. The timeseries contains 107 tracks. Note that SAM currently doesn't support automatic tracking, which is a disadvantage compared to TrackMate. We plan to support this feature in the future. In addition, note that TrackMate relies on initial segmentation from StarDist (it also supports ilastik or CellPose) and does not enable model finetuning. For the data used here the StarDist segmentation is good enough to not impede the tracking result, but for more difficult cases where the segmentation problem is not solved to such a high quality, this fact could be a disadvantage compared to µSAM. We have validated the tracking annotations obtained with all four approaches using the tracking score (TRA) defined in the Cell Tracking Challenge (see Inference and Evaluation for details, higher scores are better and 1.0 is best). The scores are 0.984 for “µSAM (Default)”, 0.982 for “µSAM (Generalist)”, 0.973 for “µSAM (Finetuned)” and 0.950 for “TrackMate (Stardist)”. The scores for µSAM are all comparable and better than TrackMate. Given the limited nature of this study we don’t want to claim a general advantage of our approach here, more experiments would be needed for this.

Supplementary Videos

Supplementary Video 1: Quick-start video that explains the installation and how to get started. The video is available at https://youtu.be/gcv0fa84mCc.

Supplementary Video 2: Tutorial for the 2D annotation tool. The video is available at https://youtu.be/9xjJBg_Bfuc.

Supplementary Video 3: Tutorial for the 3D annotation tool. The video is available at https://youtu.be/nqpyNQSyu74.

Supplementary Video 4: Tutorial for the tracking annotation tool. The video is available at https://youtu.be/1gg8OPHqOyc.

Supplementary Video 5: Tutorial for the image series annotation tool. The video is available at https://youtu.be/HqRoImdTX3c.

Supplementary Tables

**Dataset**	**Image Modality**	**Annotations**	**Source**	**Used for**	**Size in GB | Num. Samples (Avg. Dimension: Y x X, Z x Y x X)**
LIVECell	Phase-Contrast Microscopy	Cells	Edlund *et al.*[25]	Generalist Training and Evaluation	5.61 | 5239 (520, 704)
DeepBacs	Different Label-free Modalities	Bacteria	Spahn *et al.*[28]	Generalist Training and Evaluation	0.27 | 190 (771, 771)
TissueNet	Whole Tissue Imaging	Cells	Greenwald *et al.*[2]	Generalist Training and Evaluation	8.21 | 7002 (350, 350)
PlantSeg (Root)	Lightsheet Microscopy	Cells	Wolny *et al.*[30]	Generalist Training and Evaluation	2.03 | 28 (352, 503, 1310)
DSB	Fluorescence	Nuclei	Caicedo *et al.*[26]	Generalist	0.19 | 497

Nuclei	Microscopy			Training	(328, 363)
NeurIPS CellSeg	Different LM Modalities	Cells	Ma *et al.*[29]	Generalist Training	9.93 | 1151 (1040, 1118, 3)
Covid IF	Immuno-fluorescence	Cells	Pape *et al.*[32]	Generalist Evaluation	0.49 | 49 (1001, 1024)
HPA	Confocal Microscopy	Cells	Ouyang *et al.*[61]	Generalist Evaluation	8.43 | 276 (4, 2062, 2062)
Lizard	Histopathology	Nuclei	Graham *et al.*[33]	Generalist Evaluation	0.66 | 238 (3, 934, 1055)
Mouse Embryo	Lightsheet Microscopy	Nuclei	Bondarenko *et al.*[34]	Generalist Evaluation	8.59 | 35 (120, 852, 585)
PlantSeg (Ovules)	Confocal Microscopy	Cells	Wolny *et al.*[30]	Generalist Evaluation	3.22 | 31 (317, 910, 948)
Cell Tracking Challenge	Different LM Modalities	Cells	Ulman *et al.*[31]	Generalist Training	4.05 | 7599 (Heterogeneous)
Dynamic Nuclear Net	Fluorescence Modalities	Nuclei	Schwartz *et al.*[41]	User study	2.44 | 7084 (512, 512)
PanNuke	Histopathology	Nuclei	Gamper *et al.*[65]	Generalist Evaluation	2.09 | 3 (3, 2634, 256, 256)

Supplementary Table 1: Overview of LM datasets, including imaging modality, annotated structure, corresponding publication, use within our paper and size of the dataset. The cell tracking challenge data contains a mix of different datasets, for which we do not report the average dimensions because they are too diverse.

**Dataset**	**Image Modality**	**Annotations**	**Source**	**Used for**	**Details: Size (in GB) | Num. Samples (Avg. Dimension: Y x X, Z x Y x X)**
Lucchi	FIBSEM	Mitochondria	Lucchi *et al.*[36]	Generalist Evaluation	0.21 | 2 (165, 768, 1024)
MitoLab Training	Different EM Modalities	Mitochondria	Conrad *et al.*[5]	Generalist Training	6.26 | 594 (374, 393)
MitoLab Evaluation	Different EM Modalities	Mitochondria	Conrad *et al.*[5]	Generalist Evaluation	4.12 | 106 (Heterogeneous)

MitoEM	SBEM	Mitochondria	Wei *et al.*[35]	Generalist Training and Evaluation	30.24 | 6 (334, 4096, 4096)
UroCell	FIBSEM	Mitochondria	Mekuč *et al.*[37]	Generalist Evaluation	0.17 | 8 (256, 256, 256)
VNC	TEM	Mitochondria	Gerhard *et al.*[62]	Generalist Evaluation	0.04 | 2 (20, 1024, 1024)
CREMI	TEM	Neuronal processes	Funke *et al.*[63]	Specialist Training and Evaluation	0.57 | 3 (125, 1250, 1250)
NucMM-M	High-energy X-Ray	Nuclei	Lin *et al.*[38]	Generalist Evaluation	0.03 | 8 (192, 192, 192)
PlatyEM (Nuclei)	SBEM	Nuclei	Vergara *et al.*[4]	Generalist Training and Evaluation	2.86 | 12 (130, 442, 432)
PlatyEM (Cilia)	SBEM	Cilia	Vergara *et al.*[4]	Generalist Evaluation	0.31 | 5 (98, 899, 816)
ASEM (Mito)	FIBSEM	Mitochondria	Gallusser *et al.*[40]	Generalist Evaluation	19.1 | 6 (1150, 772, 2566)
ASEM (ER)	FIBSEM	Endoplasmic Reticulum	Gallusser *et al.*[40]	Specialist Training and Evaluation	18.13 | 5 (1099, 647, 2537)
SpongeEM	FIBSEM	Cells, cilia and microvilli	Musser *et al.*[64]	Generalist Evaluation	0.16 | 3 (96, 896, 896)

Supplementary Table 2: Overview of EM datasets, including imaging modality, annotated structure, corresponding publication, use within our paper and size of the dataset. The MitoLab evaluation data contains a mix of different datasets, for which we do not report the average dimensions because they are too diverse.

Supplementary Figures

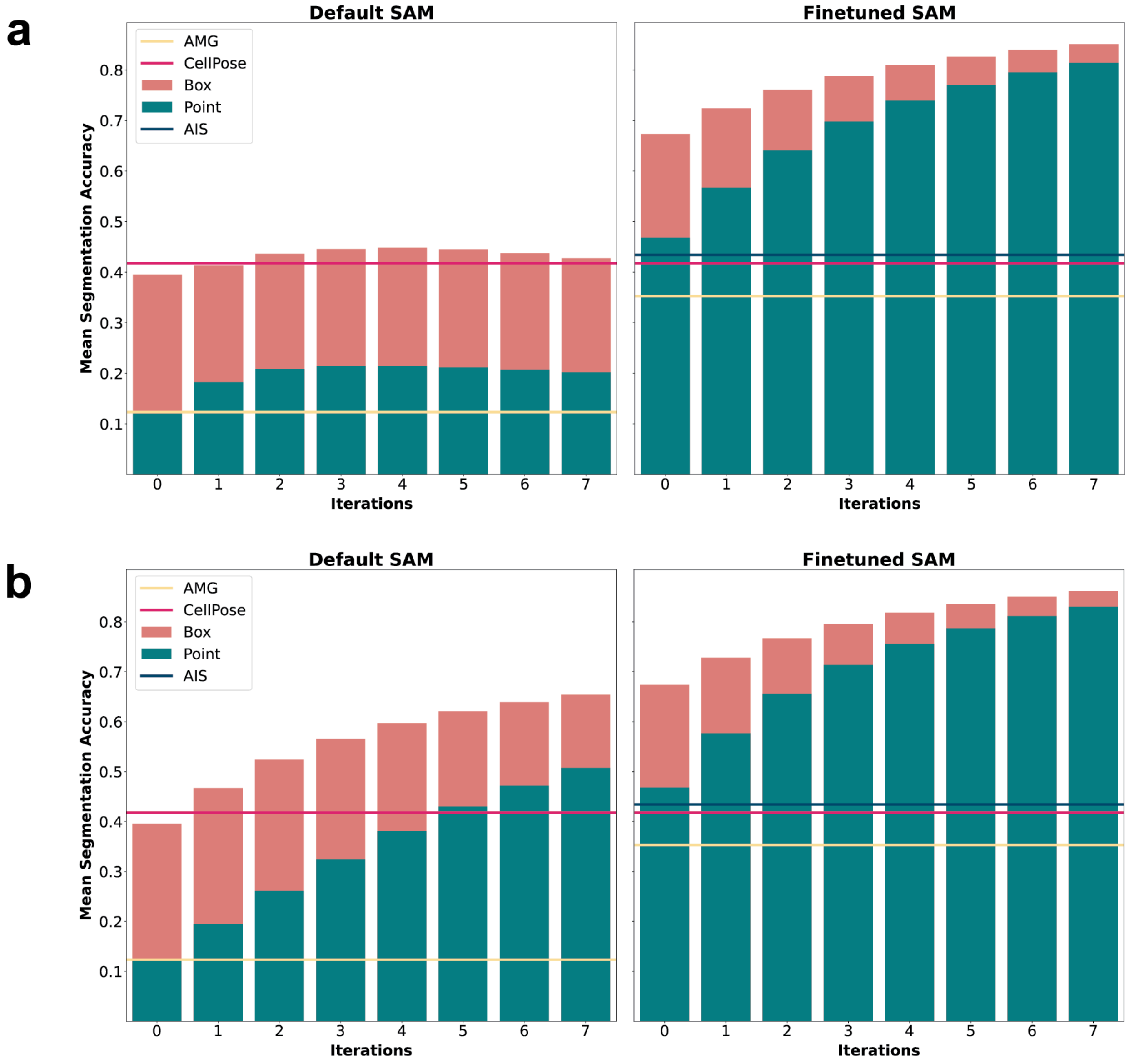

Supplementary Figure 1: Comparison of interactive segmentation without (**a**) and with (**b**) use of the previous segmented mask as additional prompt. We compare the default and finetuned ViT-L model on LIVECell, the experimental set-up corresponds to Fig. 2 a. For **b** we update the interactive segmentation evaluation to also use the previous output of the model as prompt in the next iteration. We perform this experiment to investigate a difference between our and the original SAM training scheme: we only use the mask prompt with a 50% probability during training whereas the original seems to always use it (according to the description in Kirilov et *al.*, which is consistent with the results here). We find that the original model only improves consistently with corrections when the additional mask prompt is used, whereas our model improves consistently in both settings. The set-up used for interactive segmentation for all other

experiments corresponds to **a** because this is how we have implemented interactive annotation in our napari tools. Using mask prompts would complicate the implementation significantly.

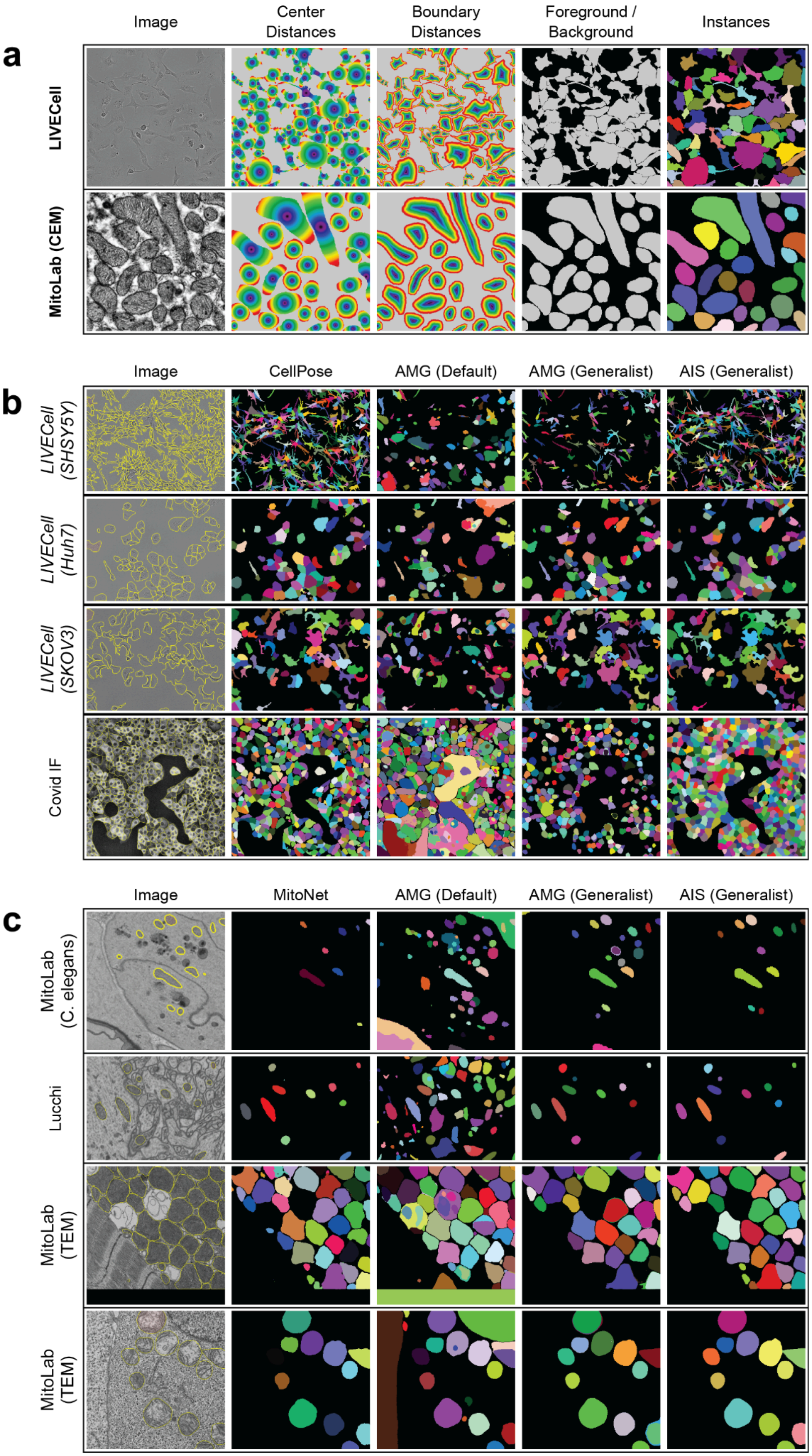
a
Image
Center Distances
Boundary Distances
Foreground / Background
Instances
LIVECell
MitoLab (CEM)
b
Image
CellPose
AMG (Default)
AMG (Generalist)
AIS (Generalist)
LIVECell (SHSY5Y)
LIVECell (Huh7)
LIVECell (SKOV3)
Covid IF
c
Image
MitoNet
AMG (Default)
AMG (Generalist)
AIS (Generalist)
MitoLab (C. elegans)
Lucchi
MitoLab (TEM)
MitoLab (TEM)

Supplementary Figure 2: Automatic segmentation implementation and results. **a.** Targets for center and boundary distances as well as foreground map used for AIS training, shown for two example images. **b**. Automatic segmentation results for LIVECell and Covid IF, comparing CellPose with AMG for the default SAM and our LM generalist model as well as AIS for the generalist. **c** shows a similar comparison for mitochondrion segmentation, with MitoNet reference results.

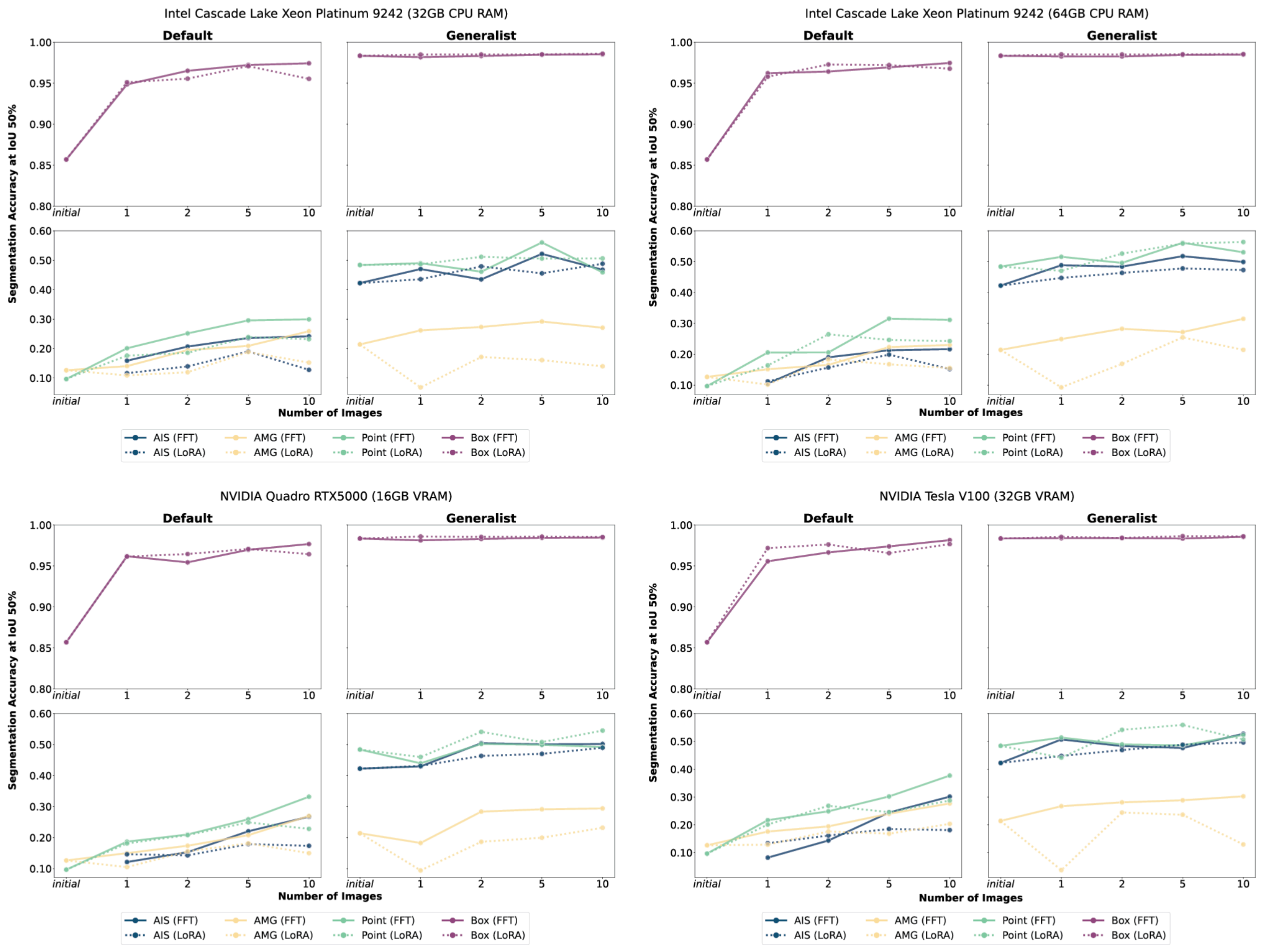

Supplementary Figure 3: Resource efficient finetuning on Covid IF dataset for four different hardware configurations. The overall experimental set-up corresponds to Fig. 5 b. Dashed lines indicate parameter-efficient training with LoRA using rank 4. The corresponding settings are given in Supp. Fig. 12 c and the training times in Supp. Fig. 12 d. We do not see significant differences between the segmentation quality between the finetuning settings for this experiment. But note that training on a GPU is much faster (Supp. Fig. 12 d).

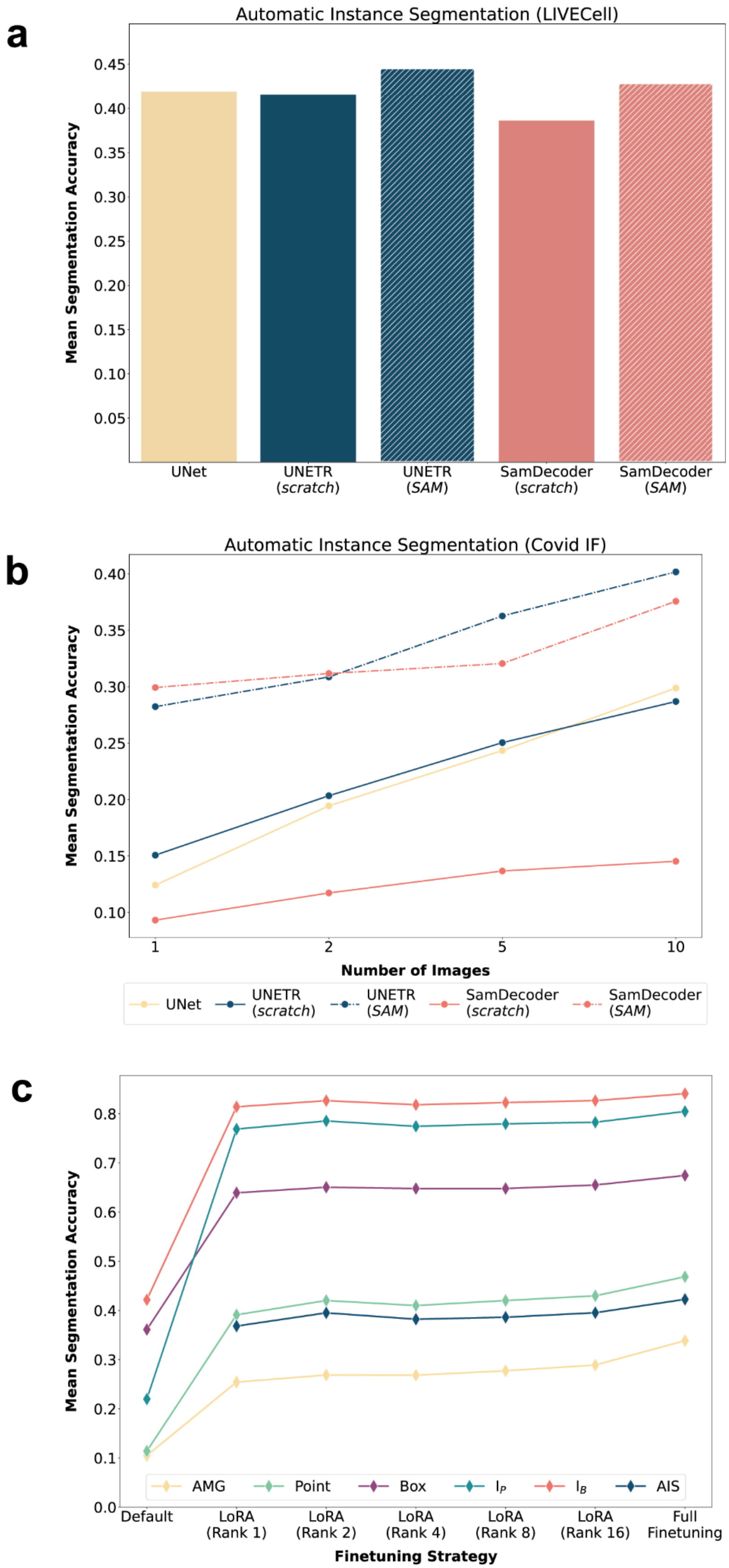
a
Automatic Instance Segmentation (LIVECell)
Mean Segmentation Accuracy
0.45
0.40
0.35
0.30
0.25
0.20
0.15
0.10
0.05
UNet
UNETR (*scratch*)
UNETR (*SAM*)
SamDecoder (*scratch*)
SamDecoder (*SAM*)
b
Automatic Instance Segmentation (Covid IF)
Mean Segmentation Accuracy
0.40
0.35
0.30
0.25
0.20
0.15
0.10
1
2
5
10
Number of Images
UNet
UNETR (*scratch*)
UNETR (*SAM*)
SamDecoder (*scratch*)
SamDecoder (*SAM*)
c
Mean Segmentation Accuracy
0.8
0.7
0.6
0.5
0.4
0.3
0.2
0.1
0.0
AMG
Point
Box
I_P
I_B
AIS
Default
LoRA (Rank 1)
LoRA (Rank 2)
LoRA (Rank 4)
LoRA (Rank 8)
LoRA (Rank 16)
Full Finetuning
Finetuning Strategy

Supplementary Figure 4: Comparison of different architectures for instance segmentation and LoRA ranks. **a** comparison of five different set-ups for automatic instance segmentation trained on the LIVECell training split and evaluated on its test split. We compare a 2D UNet[62] (64 initial channels which are doubled across four downsampling layers) with four different set-ups that build on top of the SAM architecture: UNETR[48] denotes the SAM image encoder followed by a convolutional decoder. SamDecoder denotes the SAM image encoder followed by the SAM mask decoder. We use ViT-B as image encoder for the SAM derived architectures. For the *(scratch)*-models the weights of the image encoder are randomly initialized and for the *(SAM)*-models they are initialized with the default SAM weights; for SamDecoder the decoder weights are also initialized with the model weights in this case. We predict a foreground and two distance channels with all five models, and then follow the instance segmentation procedure outlined in Methods. The results show comparable segmentation quality for all five models, with a slight advantage for SAM initialized models and a slight advantage of UNETR over SamDecoder. **b** comparison of architectures for automatic instance segmentation on Covid-IF. We use the same model set-up as in **a** and the same data splits (1, 2, 5, 10 training images) as in Fig. 5. Here, we see that the SAM initialized models perform clearly better, and that UNETR is better than SamDecoder. These results highlight the value of weight initialization for small training data and our decoder architecture choice. **c** comparison of LoRA ranks on the LIVECell dataset. Training and evaluation set-up correspond to Fig. 2 a and other LIVECell experiments. We vary the rank of projection tensors used for parameter reduction in LoRA from 1 to 16, see the LoRA publication[46] for details on the low-rank tensor decomposition. “Default” indicates the pre-trained SAM and “Full Finetuning” indicates updating all weights (the standard training strategy used in all experiments not involving LoRA). The experiment is done with ViT-B . Overall, we see that the segmentation quality increases modestly with LoRA rank and that full finetuning provides modest advantages over LoRA. However, given that LoRA does not provide a consistent improvement in training times (see Supp. Fig. 12 d), we do not use LoRA by default when finetuning models.

Additional References

The references here refer to the same numbering as in the main manuscript. Here, we only provide additional references that are used in the supplementary material, but are not used in the main manuscript.

61. Ouyang, W. *et al.* Analysis of the Human Protein Atlas Image Classification competition. *Nat. Methods* **16**, 1254–1261 (2019).

62. Gerhard, S. *et al.* Segmented anisotropic ssTEM dataset of neural tissue. figshare. http://dx.doi.org/10.6084/m9.figshare.856713 (2013).

63. Funke, J. *et al.* *MICCAI Challenge on Circuit Reconstruction from Electron Microscopy*

Images. *https://cremi.org/* *(2016).*

64. Musser, Jacob M. *et al.* Profiling cellular diversity in sponges informs animal cell type and nervous system evolution. *Science* 374.6568 (2021): 717-723. https://doi.org/10.1126/science.abj2949

65. Gamper, J. *et al.* PanNuke: An Open Pan-Cancer Histology Dataset for Nuclei Instance Segmentation and Classification. *Digital Pathology: 15th European Congress, ECDP 2019, Warwick, UK, April 10-13, 2019, Proceedings 15. Springer International Publishing, 2019.* https://doi.org/10.1007/978-3-030-23937-4_2

66. Beier, T. *et al.* Multicut brings automated neurite segmentation closer to human performance. *Nat. Methods* **14**, 101-102 (2017).
