# Supplementary material for: Segment Anything for Microscopy
Source: Nat Methods. 2025 Feb 12;22(3):579–91. doi: 10.1038/s41592-024-02580-4 (PMC11903314; doi:10.1038/s41592-024-02580-4)
Supplement: Supplementary file 2 — Reporting Summary [file 41592_2024_2580_MOESM2_ESM.pdf]

nature portfolio

Corresponding author(s): Constantin Pape

Last updated by author(s): November 4th, 2024

Reporting Summary

Nature Portfolio wishes to improve the reproducibility of the work that we publish. This form provides structure for consistency and transparency in reporting. For further information on Nature Portfolio policies, see our Editorial Policies and the Editorial Policy Checklist.

Statistics

For all statistical analyses, confirm that the following items are present in the figure legend, table legend, main text, or Methods section.

n/a	Confirmed	
☐	☒	The exact sample size (*n*) for each experimental group/condition, given as a discrete number and unit of measurement
☒	☐	A statement on whether measurements were taken from distinct samples or whether the same sample was measured repeatedly
☒	☐	The statistical test(s) used AND whether they are one- or two-sided<br>*Only common tests should be described solely by name; describe more complex techniques in the Methods section.*
☒	☐	A description of all covariates tested
☒	☐	A description of any assumptions or corrections, such as tests of normality and adjustment for multiple comparisons
☐	☒	A full description of the statistical parameters including central tendency (e.g. means) or other basic estimates (e.g. regression coefficient) AND variation (e.g. standard deviation) or associated estimates of uncertainty (e.g. confidence intervals)
☒	☐	For null hypothesis testing, the test statistic (e.g. *F*, *t*, *r*) with confidence intervals, effect sizes, degrees of freedom and *P* value noted<br>*Give P values as exact values whenever suitable.*
☒	☐	For Bayesian analysis, information on the choice of priors and Markov chain Monte Carlo settings
☒	☐	For hierarchical and complex designs, identification of the appropriate level for tests and full reporting of outcomes
☒	☐	Estimates of effect sizes (e.g. Cohen's *d*, Pearson's *r*), indicating how they were calculated

Our web collection on statistics for biologists contains articles on many of the points above.

Software and code

Policy information about availability of computer code

Data collection	We have created a new open source software tool for microscopy image segmentation. The software is available at https://github.com/computational-cell-analytics/micro-sam and documented at https://computational-cell-analytics.github.io/micro-sam/micro_sam.html. This includes installation instructions with all required dependencies.
Data analysis	We rely on the scientific python tools for analysis of our experiment results and plotting, in particular numpy (1.26), pandas (2.1) and matplotlib (3.8). The version numbers given in parenthesis are for the main python environment where the analysis was run. Note that these software packages are stable so the analysis output is expected to be stable across versions.

For manuscripts utilizing custom algorithms or software that are central to the research but not yet described in published literature, software must be made available to editors and reviewers. We strongly encourage code deposition in a community repository (e.g. GitHub). See the Nature Portfolio guidelines for submitting code & software for further information.

Data

Policy information about availability of data

All manuscripts must include a data availability statement. This statement should provide the following information, where applicable:

- Accession codes, unique identifiers, or web links for publicly available datasets
- A description of any restrictions on data availability
- For clinical datasets or third party data, please ensure that the statement adheres to our policy

Our software is available on github under a permissive open source license at https://github.com/computational-cell-analytics/micro-sam. It is documented at https://computational-cell-analytics.github.io/micro-sam/micro_sam.html. The version at submission of this manuscript is 1.1.1. Our LM and EM generalist models are available on BioImage.IO and Zenodo. Please refer to our model documentation for the ids and dois of the individual models. Additional models are deposited on Zenodo and the corresponding links are given in the documentation. The tables and code for generating quantitative plots are available on github. We make use of publicly available datasets for most experiments. They are listed in Supp. Table 1 and 2. We use new datasets for the 2D and 3D annotation user studies. These datasets are avaialble on zenodo, please see the data availability section for their DOIs.

Human research participants

Policy information about studies involving human research participants and Sex and Gender in Research.

Reporting on sex and gender	NA
Population characteristics	NA
Recruitment	NA
Ethics oversight	NA

Note that full information on the approval of the study protocol must also be provided in the manuscript.

Field-specific reporting

Please select the one below that is the best fit for your research. If you are not sure, read the appropriate sections before making your selection.

☒ Life sciences ☐ Behavioural & social sciences ☐ Ecological, evolutionary & environmental sciences

For a reference copy of the document with all sections, see nature.com/documents/nr-reporting-summary-flat.pdf

Life sciences study design

All studies must disclose on these points even when the disclosure is negative.

Sample size	We perform different kinds of experiments, with different definitions of sample size. To evaluate segmentation algorithms on existing datasets we use approximately 30 different datasets. Here, a larger size is desirable to provide a coverage of relevant experimental settings, but requires additional experimental and computational effort. In the user study we provide a multi annotator study with 5 subjects to investigate inter annotator effects. There are no clear statistical criteria for the number of annotators, five was the number we could recruit and coordinate among the co-authors.
Data exclusions	We did not exclude any data from experiments.
Replication	We performed replications for one of the segmentation evaluation experiments (Extended Data Figure 1) to investigate the effect of randomness in the interactive segmentation procedure. We tested this for five independent replications.
Randomization	Randomization of experiments is not applicable for our study.
Blinding	Blinding of experiments is not applicable for our study.

Reporting for specific materials, systems and methods

We require information from authors about some types of materials, experimental systems and methods used in many studies. Here, indicate whether each material, system or method listed is relevant to your study. If you are not sure if a list item applies to your research, read the appropriate section before selecting a response.

Materials & experimental systems

n/a	Involved in the study
☒	☐ Antibodies
☒	☐ Eukaryotic cell lines
☒	☐ Palaeontology and archaeology
☒	☐ Animals and other organisms
☒	☐ Clinical data
☒	☐ Dual use research of concern

Methods

n/a	Involved in the study
☒	☐ ChIP-seq
☒	☐ Flow cytometry
☒	☐ MRI-based neuroimaging
